# Impact of endogenous glucocorticoid on response to immune checkpoint blockade in patients with advanced cancer

**DOI:** 10.3389/fimmu.2023.1081790

**Published:** 2023-04-11

**Authors:** Yu Cui, Xinyue Han, Hongtao Liu, Qi Xie, Yaping Guan, Beibei Yin, Junjuan Xiao, Dongfeng Feng, Xuan Wang, Junwei Li, Jinghua Chen, Xiaolin Liu, Xingyu Li, Weiwei Nie, Lin Ma, Hairong Liu, Jing Liang, Yan Li, Baocheng Wang, Jun Wang

**Affiliations:** ^1^ Department of Oncology, The First Affiliated Hospital of Shandong First Medical University and Shandong Provincial Qianfoshan Hospital, Jinan, China; ^2^ Shandong Lung Cancer Institute, Jinan, China; ^3^ Shandong Key Laboratory of Rheumatic Disease and Translational Medicine, Jinan, China; ^4^ Department of Pathology, The First Affiliated Hospital of Shandong First Medical University and Shandong Provincial Qianfoshan Hospital, Jinan, China; ^5^ Department of Oncology, The 960th Hospital, The PEOPLE’s Liberation Army, Jinan, China

**Keywords:** glucocorticoid, programmed cell death protein-1, programmed cell death ligand-1, immune checkpoint inhibitor, advanced cancer

## Abstract

**Background:**

Previous studies indicate that exogenous use of glucocorticoid (GC) affects immune checkpoint inhibitor (ICI) efficacy. However, there is a paucity of clinical data evaluating the direct impact of endogenous GC on the efficacy for cancer patients with immune checkpoint blockade.

**Methods:**

We first compared the endogenous circulating GC levels in healthy individuals and patients with cancer. We next retrospectively reviewed patients with advanced cancer with PD-1/PD-L1 inhibitor alone or combination therapy in a single center. The effects of baseline circulating GC levels on objective response rate (ORR), durable clinical benefit (DCB), progression‐free survival (PFS), and overall survival (OS) were analyzed. The association of the endogenous GC levels with circulating lymphocytes, cytokines levels, and neutrophil to lymphocyte ratio, and tumor infiltrating immune cells, were systematically analyzed.

**Results:**

The endogenous GC levels in advanced cancer patients were higher than those in early-stage cancer patients as well as healthy people. In the advanced cancer cohort with immune checkpoint blockade (n=130), patients with high baseline endogenous GC levels (n=80) had a significantly reduced ORR (10.0% *vs* 40.0%; p<0.0001) and DCB (35.0% *vs* 73.5%, p=0.001) compared to those with low endogenous GC levels (n=50). The increased GC levels was significantly associated with reduced PFS (HR 2.023; p=0.0008) and OS (HR 2.809; p=0.0005). Moreover, statistically significant differences regarding PFS, and OS were also detected after propensity score matching. In a multivariable model, the endogenous GC was identified as an independent indicator for predicting PFS (HR 1.779; p=0.012) and OS (HR 2.468; p=0.013). High endogenous GC levels were significantly associated with reduced lymphocytes (p=0.019), increased neutrophil to lymphocyte ratio (p=0.0009), and increased interleukin-6 levels (p=0.025). Patients with high levels of endogenous GC had low numbers of tumor infiltrating CD3^+^ (p=0.001), CD8^+^ T (p=0.059), and CD4^+^ T (p=0.002) cells, and the numbers of circulating PD-1^+^ NK cells (p=0.012), and the ratio of CD8^+^PD-1^+^ to CD4^+^PD-1^+^ (p=0.031) were higher in patients with high levels of endogenous GC compared to low levels of endogenous GC.

**Conclusion:**

Baseline endogenous GC increase executes a comprehensive negative effect on immunosurveillance and response to immunotherapy in real-world cancer patients accompanied with cancer progression.

## Background

Immune checkpoint blockade using immune checkpoint inhibitors (ICIs), including cytotoxic T lymphocyte-associated antigen-4 (CTLA-4) and programmed cell death protein-1 (PD-1)/programmed cell death ligand-1 (PD-L1) inhibitors, has revolutionized the systematic treatment for various malignancies at the advanced or metastatic stage, with unprecedented survival benefit and tolerable toxicity. ICIs selectively restore and normalize the body’s antitumor immune responses by disrupting the immunoinhibitory signals mediated by the PD-1/PD-L1 and CTLA-4 axes in the tumor microenvironment (1). Nivolumab, pembrolizumab, cemiplimab, avelumab, durvalumab, atezolizumab, and ipilimumab are currently approved standards of care and have shifted the treatment paradigm for certain human malignancies, including previously treated or untreated non-small cell lung cancer (NSCLC), melanoma, and other human solid tumors (2). PD-L1 expression on tumor cells is initially characterized as a biomarker for predicting the response to ICI therapy (3). Other tumor factors related to enhanced clinical benefit from immunotherapy include deficient mismatch repair (dMMR) protein, high microsatellite instability (MSI-H), high tumor mutational burden (TMB), and the effector T-cell gene signature. However, tumoral PD-L1 positivity is insufficient for selecting patients benefiting from immunotherapy because patients with negative PD-L1 expression or low TMB are also responsive to immunotherapy (4). Tumor-derived PD-L1 and other biomarkers have some limitations, involving technical difficulties, invasive feature because of the need for tumor biopsy, and the lack of consideration of comprehensive host immune status (5). Recent studies have indicated that baseline circulating predictors from the host, including CD8^+^PD-1^+^ T cells, serum tumor markers, neutrophil to lymphocyte ratio (NLR), and diversity of gut microbiome, can aid in evaluating the therapeutic outcomes of ICI treatment (6–9).

Glucocorticoid (GC) has anti-inflammatory, anti-shock, and immunosuppressive properties (10). Although low-dose dexamethasone at the initial stage of treatment is helpful to improve the efficacy of anti-PD-1 treatment in cancer by suppressing immune evasion (11, 12), but high-dose synthetic GCs such as dexamethasone and prednisolone are found to suppress T cell proliferation and function and decrease response to anti-PD-1 and anti-CTLA-4 immune checkpoint blockade *in vitro and* animal experiments (13, 14). GC can potentiate the inhibitory capacity of PD-1 by up-regulating its expression on tumor-infiltrating T and NK cells (15). Exogenous GC is widely used in the management of cancer patients with a variety of clinical conditions, including dyspnea, fatigue, lack of appetite, and symptomatic brain metastases with edema. GC is also first-line agent against immune-related adverse events (irAEs) that may develop following immunotherapy, particularly immune checkpoint blockade (16, 17). The baseline use of GC at a high dose of ≥10 mg of prednisone equivalent daily for palliative indications is associated with poor outcome in NSCLC patients with PD-1/PD-L1 blockade (18, 19). Even early use of high dose of GC for treating irAEs after the initiation of anti-PD-1 monotherapy is associated with poor survival (20). Furthermore, patients treated with glucocorticoid ≥2 months after starting immunotherapy had a statistically significant longer progression-free survival (PFS), and overall survival (OS) than those who received glucocorticoid <2 months after starting immunotherapy (21).

By contrast, endogenous GC cortisol secretion in human occurs in response to hyperactivity of the hypothalamic-pituitary-adrenal (HPA) axis during physical and psychological stress, and play an important role in disease development, especially cancer progression when the diagnosis of cancer has been already made (22, 23). Stress can induce circulating GC surge and Tsc22d3 upregulation, which subvert anticancer therapy-induced immunosurveillance and abolish therapeutic control of tumors including chemotherapy and immunotherapy (24). Neuroendocrine alterations including a dysregulation of endogenous GC secretion and serum cortisol rhythm usually occurred in patients with advanced cancer and is associated with advanced cancer stage poor clinical outcome (25–29). High random serum cortisol level is found to be an independent predictor of OS for terminally ill cancer patients (30). However, there is a paucity of clinical data evaluating the direct effect of endogenous GC on ICI efficacy for cancer patients with immune checkpoint blockade. In this study, we therefore evaluated whether endogenous GC at the initiation of ICIs may impair the efficacy of PD-1/PD-L1 inhibitors in patients with advanced or metastatic cancer, and further explored whether endogenous GC levels are associated with circulating and tumor-infiltrating T cell subpopulations, and cytokine secretion.

## Materials and methods

### Peripheral blood mononuclear cells isolation and agents

Peripheral blood was collected at baseline into heparinized tubes for cancer patient with immunotherapy, and PBMCs were separated by Ficoll-Paque density gradient. Firstly, the blood was diluted with 1:1 volume of PBS, these suspensions were then added gently onto the Ficoll-Paque™ PLUS (17–1440–02) (GE Healthcare) from the edge of the falcon tube. The mixture was centrifuged at 2000 rpm SOFT RCC/DEC for 30 minutes. The obtained mononuclear cell layer was gently rinsed by adding in PBS, then centrifuged at 1200 rpm for 10 minutes. The cells were resuspended in CELLSAVING (Serum-free, animal protein-free Cell Freezing Medium) (NCM Biotech) at -80 °C until the flow cytometric.

### Cancer patients and healthy populations

A retrospective cohort included patients with a histologically or cytologically proven diagnosis of advanced or metastatic cancer who presented to The First Affiliated Hospital of Shandong First Medical University from December 2019 to April 2022. The inclusion criteria were as follows: age older than 18 years; histologically or cytologically confirmed locally advanced or metastatic cancer according to the American Joint Committee on Cancer (AJCC) staging system, 8th version; receiving at least two cycles of PD-1/PD-L1 inhibitor (nivolumab, pembrolizumab, camrelizumab, sintilimab, tislelizumab, durvalumab, or atezolizumab) monotherapy or a combination with chemotherapy as a first-, second- or later line of treatment for at least one tumor evaluation by imaging; an Eastern Cooperative Oncology Group (ECOG) performance status (PS) of 0–2. Patients who did not undergo re-staging imaging while on treatment, having basic cortisol deficiency, adrenalectomy, or previous radiotherapy on adrenal glands, and having a history of immunotherapy including ICIs, were excluded. Efficacy evaluation by radiographic findings was performed according to Response Evaluation Criteria in Solid Tumors version 1.1 (RECIST 1.1) and included complete response (CR), partial response (PR), objective response rate (ORR), and disease control rate (DCR). To compare the endogenous circulating GC levels in populations with versus without cancer, 61 healthy volunteers and 44 early-stage cancer patients who completed radical surgery in this center were included in this study.

Data were gathered through the electronic medical record. The clinical characteristics of the patients included age, sex, smoking status, body mass index (BMI), histology, and tumor node metastasis (TNM) stage at the start of treatment. Baseline plasma GC cortisol (normal range: 172 to 497 nmol/L) and adrenocorticotropic hormone (ACTH) (8:00 AM) levels in healthy volunteers and cancer patients were captured and recorded prior to antitumor treatment. Endogenous GC cortisol and ACTH concentrations were determined by an automated analyzer (The cobas 8000 modular analyzer series) with an ECLIA method (Roche Diagnostics, Mannheim, Germany). The NLR was calculated by dividing the absolute neutrophil counts by the lymphocyte counts, as measured in peripheral blood. This study was approved by the independent research ethics committee of The First Affiliated Hospital of Shandong First Medical University (NO: YXLL-KY-2020–007) and conformed to the principles of the Declaration of Helsinki.

### Flow cytometry analysis

APC anti-human CD8 (SK1), FITC anti-human CD4 (OKT4), FITC anti-human CD16 (3G8), APC anti-human CD56 (NCAM) (HCD56), PE/Dazzle™594 anti-human CD279 (PD-1) (EH12.2H7), were purchased from BioLegend. Cells were stained for 30 min at room temperature, rinsed with FACS buffer (1% BSA and 0.01% sodium azide in PBS), fixed with 4% paraformaldehyde (Alfa Aesar, WA, USA), and resuspended in FACS buffer for flow cytometry analysis. Samples run on a BD LSR Fortessa using BD FACSDiva software (BD Biosciences). Data was analyzed using FlowJo V10 (Tree Star Inc) and GraphPad prism 9.0.

### Enzyme-linked immunosorbent assay

Cytokines including interleukin (IL)-2, IL-4, IL-6, IL-10, interferon-γ (IFN-γ), and tumor necrosis factor-α (TNF-α) were detected using Human Cytokine Standard Assays panel (ET Healthcare, Inc., Shanghai, China) and the Bio-Plex 200 system (Bio-Rad, Hercules, CA, USA) according to the manufacturer’s instructions.

### Immunohistochemical analysis

IHC staining was performed according to the previous procedures (31). Briefly, 4-μm-thick sections of tumor tissues were cut from FFPE blocks and mounted on slides. All slides were dried for 2 h at 62 °C. Sections were subsequently deparaffinized in xylene and rehydrated in graded alcohol. Antigen retrieval was achieved by heating the slides in a target retrieval solution (pH 6.0; 0.01 mol/L citrate buffer) for 15 min, then cooling them for 90 min at room temperature. After the endogenous peroxidase activity was blocked by incubation with 3% hydrogen peroxide in methanol for 10 min, nonspecific binding was blocked by incubating the slides with 5% bovine serum albumin in phosphate-buffered saline (PBS) for 30 min. After the specimens were washed three times with PBS, they were reacted overnight at 4 °C with primary mouse anti-human monoclonal CD8, CD4, and CD3 antibody (1:200 solution; Santa Cruz Biotechnology, Santa Cruz, CA, USA). After incubation with a biotin-conjugated secondary antibody for 30 min at room temperature, sections were further treated with an avidin-biotin-peroxidase complex system (RTU VECTASTAIN kit, VECTOR LABORATORIES, Burlingame, CA, USA). Finally, the signal was developed with 3,3´-diaminobenzidine tetrahydrochloride (1:50 solution; DAB Substrate kit, Abcam, Cambridge, MA, USA). All sections were then counterstained with hematoxylin and mounted.

### Multiple immunofluorescences

Tumor tissues were obtained by operation, fixed in formalin and embedded in paraffin. For the Panck/Foxp3/PD-1/CD4/CD8 multiplex panel, a cocktail of primary antibodies including Panck, Foxp3, PD-1, CD4, and CD8 were used, Immunofluorescence staining was performed according to standard procedures (Akoya Biosciences, NEL871001KT). Briefly, paraffin sections were repaired using sodium citrate for 10-25 min, blocked using BF block buffer containing 30%-40% goat serum for 1 hour at room temperature, then incubated with primary antibodies against Panck (Zsgb.bio, catalog number zm0069, 1:200 dilution), Foxp3 (Abcam, catalog number ab20034, 1:100 dilution), PD1 (Zsgb.bio, catalog number zm0381, stock solution), CD4 (Zsgb.bio, catalog number zm0418, stock solution), CD8 (Abcam, catalog number ab199016, 1:500 dilution) at 4°C overnight with additional 2 h at room temperature, and then incubated with secondary antibodies for 10 min followed by appropriate opal fluorophore (690, 520, 570, 480 and 780, respectively) reagent at room temperature for 10min. Finally, the paraffin sections were stained with DAPI (1:500 dilution) for 7 min at room temperature and subjected to standard analysis by Halo Link software (Indica Labs).

### Statistical analysis

Categorical variables, such as patients’ demographics, disease characteristics, and medical history, were reported as frequencies and percentages. Quantitative variables are presented as medians and ranges. For categorical data, χ^2^ or Fisher-exact test was used to compare the groups. For continuous variables, independent sample *t*-test or Mann-Whitney U test was used to compare patient groups. The Cutoff Finder web application tool was used to fit Cox proportional hazard models to dichotomize clinicopathological variables and the survival variables. The optimal cutoff value of GC level (low and high GC group) was defined as the point with the most significant (log-rank test) split (32). In addition, low, intermediate and levels of endogenous GC are set according to calculating quartiles. Durable clinical benefit (DCB) was defined as CR and PR, or stable disease (SD) that lasted longer than 6 months otherwise patients were considered as no durable clinical benefit (NDB). PFS was defined as the time from immunotherapy initiation to the date of disease progression or death from any cause, whichever occurred first. Patients who were alive without disease progression were censored on the date of their last disease assessment. OS was defined as the time from immunotherapy initiation to death from any cause. Patients who were still alive were censored at the date of last contact. PFS and OS curves were calculated using the Kaplan–Meier method and were compared using the log-rank test. Cox proportional hazard regression methods were used to estimate the survival probability, the hazard ratio (HR) and 95% confidence interval (CI). Factors that were statistically significant in the univariate analysis were incorporated into the multivariate analysis. Propensity score matching (PSM) was used to balance the baseline characteristics between the low GC group and high GC group. thus, we used a logistic regression model to calculate the propensity score for each patient and match 1:1 for the two groups. After PSM, we used standardized mean differences to evaluate the balance of characteristics between the two groups. A SPSS 26.0 software (SPSS Inc., Chicago, IL) was used for all statistical tests. A p value <0.05 was considered statistically significant.

## Results

### The endogenous GC levels in healthy individuals, early-stage, and advanced cancer patients

**Figure 1:**
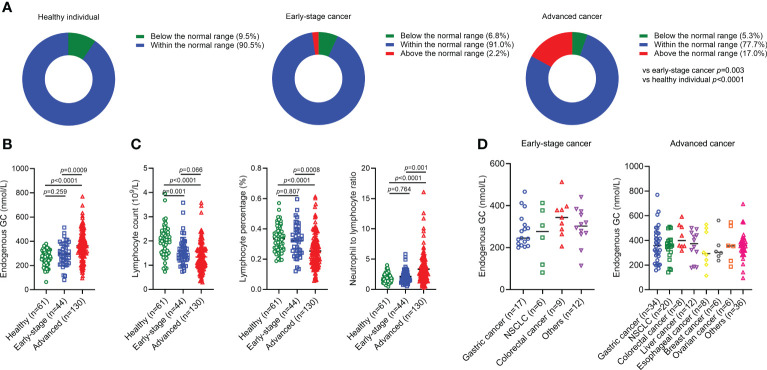
The endogenous GC levels in healthy individuals and cancer patients. **(A)** Comparing the percentage of endogenous GC levels above the normal range in healthy individuals (n=61) and cancer patients with early (n=44) and advanced (n=130) stages. **(B)** Comparing the endogenous GC levels in healthy individuals and cancer patients with different disease stage. **(C)** Comparing the absolute lymphocyte count, lymphocyte percentage, and neutrophil-to-lymphocyte ratio in healthy individuals and cancer patients with different disease stage. **(D)** The endogenous GC levels in early-stage and advanced patients with different caner types. GC, glucocorticoid; NSCLC, non-small cell lung cancer.

We first asked whether there was significant difference regarding endogenous GC levels in healthy individuals compared to cancer patients with different disease stages. There were no healthy individuals (n=61) with an endogenous GC level above the normal range (497 nmol/L), but 2.2% of early-stage cancer patients (n=44) and 17.0% of advanced cancer patients (n=130) had an endogenous GC level above the normal range (**Figure 1A**), suggesting that differences existed in the abnormality of high endogenous GC between advanced cancer patients and early-stage cancer patients (p=0.0003) or healthy populations (p<0.0001). The endogenous GC levels in advanced cancer patients were higher than that in early-stage cancer patients (p=0.0009) as well as in healthy populations (p<0.0001) (**Figure 1B**). Patients with advanced cancer also had a decreased absolute lymphocyte count, decreased lymphocyte percentage, and increased NLR, compared to patients with early-stage cancer (p=0.066, p=0.0008, p=0.001, respectively) and healthy populations (p<0.0001, p<0.0001, p<0.0001, respectively) (**Figure 1C**). However, the endogenous GC levels in patients with different cancer types were similar regardless of disease stage (**Figure 1D**).

### The endogenous GC and the efficacy of immune checkpoint blockade

We next analyzed whether baseline endogenous GC levels affected the efficacy of immunotherapy in advanced solid tumors. In the entire cohort of advanced or metastatic cancer patients, the data of 170 patients treated with PD-1/PD-L1 inhibitors were initially reviewed. Of them, 130 patients with gastric carcinoma (n=34), including NSCLC (n=20), hepatocellular carcinoma (HCC) (n=12), small cell lung cancer (n=8), and esophageal cancer (n=8), received at least two ICI infusions and were evaluable for response to immunotherapy. Patient characteristics are presented in **Table 1**. Median follow-up was 9.5 months (range 1.3 to 27.6). The median age was 62.5 years (range 22 to 86), and 70.8% were male. 23 patients received ICI monotherapy. At the end of follow-up, 99 patients had relapsed, and 53 patients had died. For the entire population, the ORR was 21.5%, DCR was 73.3%, DCB was 52.7%, and the median OS and PFS were 18.6 months (95% CI 13.8–23.4) and 4.6 months (95% CI 3.3–5.9), respectively.

**Table 1:** Baseline characteristics of included patients with advanced or metastatic cancer.

Baseline Characteristic	Low GC (<322 nmol/L) (n=50)	High GC (≥322 nmol/L) (n=80)	p
Age, range, years	62 (30–78)	64 (22–86)	0.762
<60	22 (44)	34 (42)	0.867
≥60	28 (56)	46 (58)	
Sex			0.583
Male	34 (68)	58 (73)	
Female	16 (32)	22 (27)	
ECOG PS			0.028
0–1	46 (92)	60 (75)	
≥2	4 (8)	20 (25)	
Smoking status			0.824
Ever	26 (52)	40 (50)	
Never	24 (48)	40 (50)	
BMI, kg/m^2^			0.353
Underweight (<18.5)	3 (6)	13 (16)	
Normal (18.5–24)	32 (64)	46 (58)	
Overweight (24–28)	11 (22)	17 (21)	
Obese (≥28)	4 (8)	4 (5)	
Cancer type			0.465
Gastric carcinoma	16 (32)	18 (22)	
NSCLC	6 (12)	14 (18)	
HCC	4 (8)	8 (10)	
SCLC	2 (4)	6 (8)	
Esophageal cancer	5 (10)	3 (3)	
Others	17 (34)	31 (39)	
Treatment			0.383
Monotherapy	7 (14)	16 (20)	
Combination therapy	43 (86)	64 (80)	
ICI			0.136
Anti-PD-1 antibody	38 (76)	69 (86)	
Anti-PD-L1 antibody	12 (24)	11 (14)	
PD-L1 expression			0.872
Not available	37 (74)	57 (71)	
<1%	5 (10)	6 (8)	
1–49%	8 (16)	16 (20)	
≥50%	0 (0)	1 (1)	
MSI/MMR status			0.183
Not available	38 (76)	63 (79)	
MSI-H (dMMR)	0 (0)	4 (5)	
MSI-L/MSS (pMMR)	12 (24)	13 (16)	
Distant metastasis			0.589
Non-metastatic	12 (24)	16 (20)	
Metastatic	38 (76)	64 (80)	
Brain metastasis			1.000
Yes	2 (4)	4 (5)	
No	48 (96)	76 (95)	
Adrenal gland metastasis			0.298
Yes	0 (0)	4 (5)	
No	50 (100)	76 (95)	
Line of therapy			0.230
First line	16 (32)	18 (22)	
Second line or later	34 (68)	62 (78)	
Previous surgery			0.403
Yes	30 (60)	42 (53)	
No	20 (40)	38 (47)	
Radiotherapy			0.394
Yes	11 (22)	23 (29)	
No	39 (78)	57 (71)	
NLR			0.006
<5	48 (96)	61 (76)	
≥5	2 (4)	19 (24)	
ALB, g/L			0.288
<0	19 (38)	38 (48)	
≥40	31 (62)	42 (52)	
Lymphocyte count, × 10^9^/L			0.011
<1.1	12 (24)	37 (46)	
≥1.1	38 (76)	43 (54)	
Blood glucose, mmol/L			0.523
<6.11	42 (84)	62 (79)	
≥6.11	8 (16)	16 (21)	
Cholestenone, mmol/L			1.000
<6.17	32 (94)	43 (96)	
≥6.17	2 (6)	2 (4)	
Triglyceride, mmol/L			0.861
<1.77	29 (85)	39 (87)	
≥1.77	5 (15)	6 (13)	

ALB, albumin; BMI, body mass index; ECOG, Eastern Cooperative Oncology Group; GC, glucocorticoid; HCC, hepatocellular carcinoma; ICI, immune checkpoint inhibitor; NLR, neutrophil to lymphocyte ratio; NSCLC, non-small cell lung cancer; PD-1, programmed cell death-1; PD-L1, programmed death ligand 1; PS, performance status; SCLC, small cell lung cancer.MSI-L, low microsatellite instability; MSS, microsatellite stability; pMMR, proficient mismatch repair.

**Figure 2:**
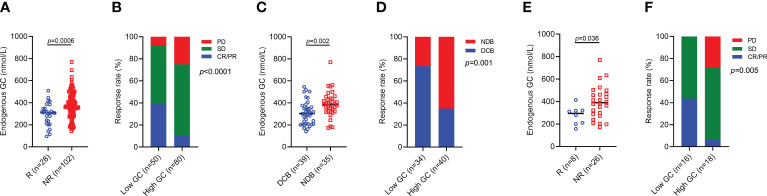
ORR and DCB for advanced cancer patients with high baseline endogenous GC levels versus low endogenous GC levels. **(A)** Comparing the baseline endogenous GC levels in all advanced cancer patients with response (R, n=28) and patients with no response (NR, n=102). **(B)** The rate of CR/PR (ORR), SD, PD in all advanced patients with high (n=80) or low (n=50) baseline endogenous GC levels. **(C)** Comparing the baseline endogenous GC levels in all advanced patients with DCB (n=39) and NDB (n=35). **(D)** The rate of DCB in all advanced patients with high (n=40) or low (n=34) endogenous GC levels. **(E)** Comparing the baseline endogenous GC levels in advanced gastric cancer patients with response (R, n=8) and patients with no response (NR, n=26). **(F)** The rate of CR/PR (ORR), SD, PD in all advanced gastric cancer patients with high (n=18) or low (n=16) baseline endogenous GC levels. GC, glucocorticoid; CR, complete response; PR, partial response; ORR, objective response rate; SD, stable disease; PD, progressive disease; DCB, durable clinical benefit; NDB, no durable clinical benefit.

We first focused on the baseline endogenous GC levels on the efficacy of ICI, and the cut-off for survival risk by log-rank maximization method was 322 nmol/L. At the time of ICI initiation, a total of 80 patients had high endogenous GC levels. Patients with response had a significantly low endogenous GC level than those with no response (p=0.0006) (**Figure 2A**). The ORR was significantly lower in the high endogenous GC group than that in the low endogenous GC group (10.0% *vs* 40.0%, p<0.0001) (**Figure 2B**). Similarly, patients with DCB had significantly higher endogenous GC levels than those with NDB) (p=0.002) (**Figure 2C**), and the difference in DCB rate between the two groups was statistically significant (35.0% *vs* 73.5%, p=0.001) (**Figure 2D**). Gastric cancer patients with response had a significantly low endogenous GC level than those with no response (p=0.036) (**Figure 2E**). High endogenous GC levels remained associated with reduced ORR in patients with gastric cancer (p=0.005), and the ORR was 6.0% and 43.7% in high and low GC levels group, respectively (**Figure 2F**).

**Figure 3:**
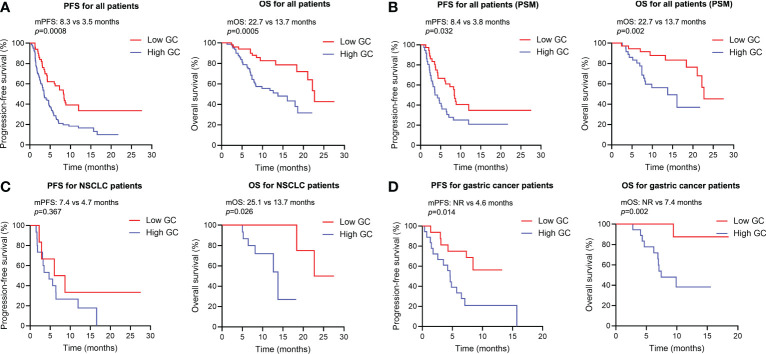
Kaplan-Meier survival curves for overall survival and progression-free survival according to the baseline endogenous GC levels. **(A)** Kaplan-Meier survival curves for progression-free and overall survival according to the baseline endogenous GC levels in all populations (n=130). **(B)** Kaplan-Meier survival curves for progression-free and overall survival according to the baseline endogenous GC levels in all populations after PSM (n=72). **(C)** Kaplan-Meier survival curves for progression-free and overall survival according to the baseline endogenous GC levels in NSCLC patients (n=20). **(D)** Kaplan-Meier survival curves for progression-free and overall survival according to the baseline endogenous GC levels in gastric patients (n=34). PSM, propensity score matching; GC, glucocorticoid; NSCLC, non-small cell lung cancer; mOS, median overall survival time; mPFS, median progression-free survival.

**Figure 4:**
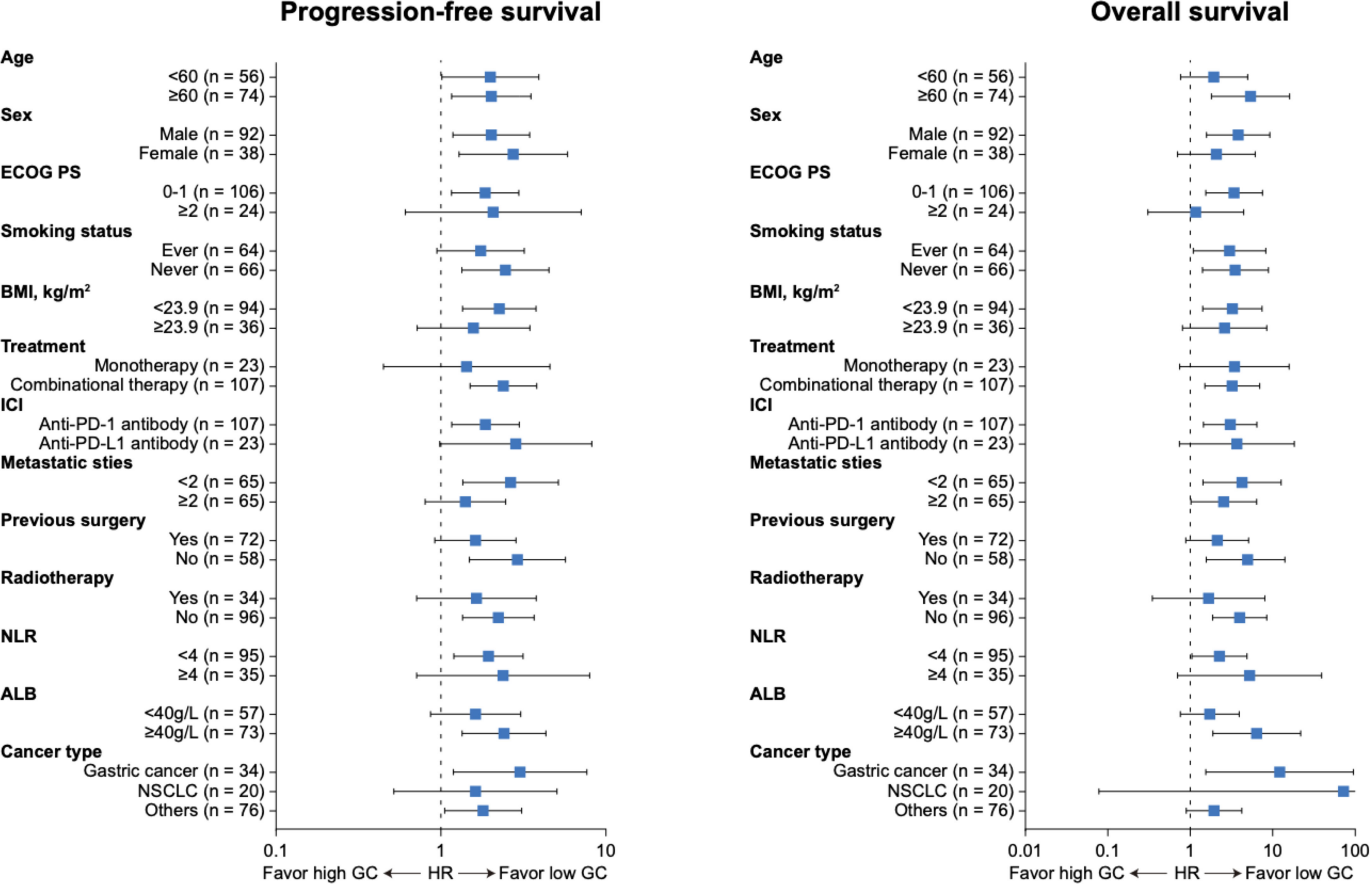
Forest plot of subgroup analyses of prognostic factors for progression-free survival and overall survival in patients with high endogenous GC levels versus low endogenous GC levels. ECOG, Eastern Cooperative Oncology Group; HR, hazard ratio; PS, performance status; GC, glucocorticoid; PD-1, programmed cell death-1; PD-L1, programmed death ligand 1; BMI, body mass index; ALB, albumin; NLR, neutrophil to lymphocyte ratio.

As for survival analysis, the patients with high endogenous GC levels had a poorer median PFS (3.5 *vs* 8.3 months; HR 2.023; 95% CI 1.364–3.001; p=0.0008) and OS (13.7 *vs* 22.7 months; HR 2.809; 95% CI 1.639–4.816; p=0.0005) than those with low endogenous GC level (**Figure 3A**). Statistically significant differences regarding PFS (HR 1.816; 95% CI 1.034–3.188; p=0.032) and OS (HR 3.084; 95% CI 1.397–6.809; p=0.002) were also detected after PSM (**Figure 3B**). The endogenous GC levels were further confirmed to stratify patients with different prognoses in terms of PFS and OS in patients with different tumor type (**Figure 3C** for NSCLC, and **Figure 3D** for gastric cancer) or different anti-cancer treatment (see **Supplemental Figure 1**). The baseline plasma cortisol levels had a consistently negative effect on the efficacy of immunotherapy, with decreased PFS and OS in other subgroups (**Figure 4**). In a multivariate analysis using Cox proportional hazards regression model, adjusting for various clinical factors, including age, smoking status, performance status, and tumor type, the endogenous GC was an independent indicator for predicting OS (HR 2.468; 95% CI 1.207–5.046; p=0.013) and PFS (HR 1.779; 95% CI 1.135–2.788; p=0.012) (**Table 2**). External low-dose dexamethasone at the initial stage of treatment has been reported to be helpful in improving the efficacy of anti-PD-1 treatment in cancer by suppressing immune evasion (11, 12), so we address the question of whether intermediate levels of endogenous GC are therapeutically more beneficial than both higher and lower levels of GC. Unfortunately, the ORR remained significantly higher in the low endogenous GC group than that in the intermediate and high endogenous GC groups (35.0% *vs* 23.0% *vs* 6.0%, p=0.043). Patients with high endogenous levels of GC had a poorer median OS (11.8 *vs* 18.6 *vs* 22.7 months; p=0.005) and PFS (2.7 *vs* 4.6 *vs* 8.2 months; p=0.006) than those with intermediate and low levels of endogenous GC level (see **Supplemental Figure 2**).

**Table 2:** Multivariable analysis of progression-free survival and overall survival.

Patient Characteristic	PFS		OS	
	HR (95% CI)	p	HR (95% CI)	p
Endogenous GC (≥322 nmol/L *vs <*322 nmol/L)	1.779 (1.135-2.788)	0.012	2.468 (1.207-5.046)	0.013
NLR (≥5 *vs <*5)	1.613 (0.954-2.727)	0.075	1.409 (0.689-2.880)	0.348
ALB (<40 g/L *vs* ≥40 g/L)	1.107 (0.728-1.683)	0.635	0.710 (0.404-1.248)	0.234
Treatment (monotherapy *vs* combination therapy)	2.193 (1.206-3.988)	0.010	0.830 (0.423-1.627)	0.587
Metastatic site (≥2 *vs <*2)	1.909 (1.264-2.884)	0.002	1.472 (0.833-2.602)	0.184
ECOG PS (≥2 *vs* 0–1)	0.523 (0.318-0.860)	0.011	0.552 (0.295-1.031)	0.062

ALB, albumin; ECOG, Eastern Cooperative Oncology Group; GC, glucocorticoid; HR, hazard ratio; NLR, neutrophil to lymphocyte ratio; PFS, progression-free survival; PS, performance status; OS, overall survival.

### The endogenous GC and circulating lymphocytes and cytokines

**Figure 5:**
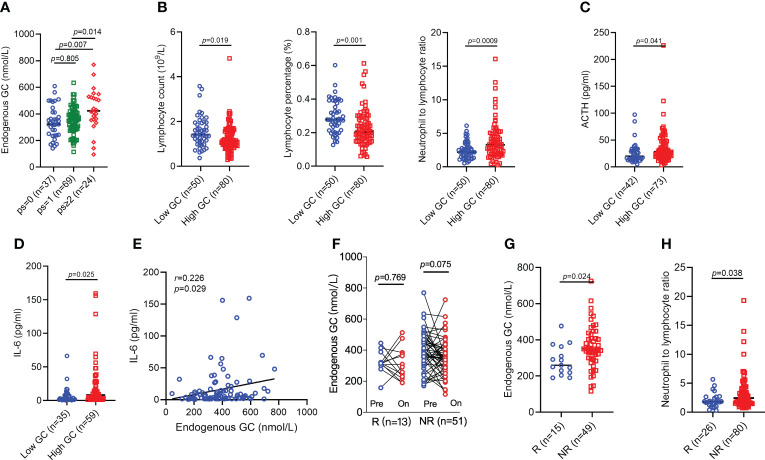
Comparing the circulating lymphocytes, cytokines levels, and ACTH levels in patients with high or low endogenous GC levels. **(A)** The baseline endogenous GC levels in patients with different ECOG PS. **(B)** The baseline absolute lymphocyte count, lymphocyte percentage, and neutrophil-to-lymphocyte ratio in cancer patients with high or low GC levels. **(C)** The ACTH levels in patients with high or low GC levels. **(D, E)** The circulating IL-6 levels in cancer patients with high or low GC levels. **(F)** Dynamics of endogenous GC (cycles 1-2) in responsive or non-responsive patients. **(G)** Comparing the endogenous GC levels in all advanced cancer patients with response and patients with no response at the time of the first evaluation (cycle 2). **(H)** Comparing the neutrophil-to-lymphocyte ratio in all advanced cancer patients with response and patients with no response at the time of the first evaluation (cycle 2). ACTH, adrenocorticotropic hormone; GC, glucocorticoid; ECOG, Eastern Cooperative Oncology Group; PS, performance status; IL-6, interleukin 6.

We next explored why the endogenous GC affected the efficacy of anti-PD-1/PD-L1 antibody immunotherapy. Clinical characteristics and some laboratory findings were typical and were generally well balanced between patients who did or did not have baseline high GC levels, with the only one expected exception; poor ECOG PS was more common in those with high cortisol levels (p=0.028) (**Table 1**). High cortisol level was also associated with increased ECOG PS (**Figure 5A**). We continued to analyze the association of the endogenous GC and circulating immune-related biomarkers. Patients with high cortisol level also had lower absolute lymphocyte count (p=0.019), lymphocyte percentage (p=0.001), and NLR by complete blood count analyzer (p=0.0009) than those with low cortisol levels (**Figure 5B**). Considering cortisol secretion is controlled by HPA axis, we also analyzed the levels of ACTH and confirmed its association with the levels of GC. High levels of ACTH were more commonly occurred in patients with high levels of GC (p=0.041) (**Figure 5C**). Using ELISA, we found that only high levels of circulating IL-6, not other five cytokines (IL-2, IL-4, IL-10, IFN-γ, and TNF-α), were more commonly found in patients with high GC levels (p=0.025) and circulating increased IL-6 levels were associated with increased GC levels (p=0.029) (**Figures 5D, E**). However, the GC levels remained no significant change after completing 2 cycles of ICI treatment (**Figure 5F**) regardless of the response to ICIs, but patients with response remained high GC levels (p=0.024) and increased NLR (p=0.038) compared to those with no response at the time of response evaluation (**Figures 5G, H**).

**Figure 6:**
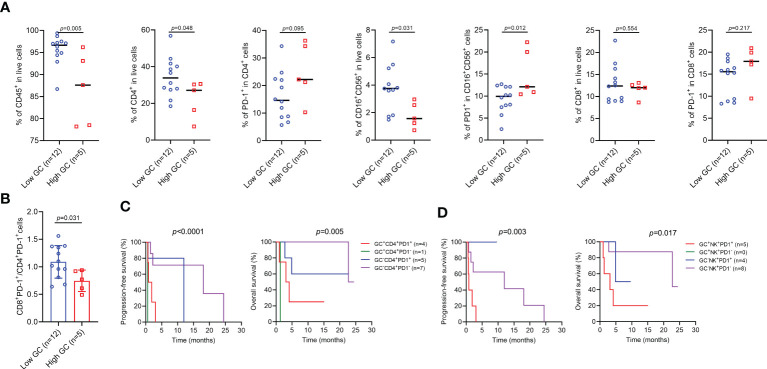
The effect of endogenous GC on the percentages of immune cell subgroups in patients with high or low endogenous GC levels. **(A)** Comparing the percentages of immune cell subgroups in patients with high (n=5) or low (n=12) endogenous GC levels. **(B)** Comparing the CD8^+^PD-1^+^ to CD4^+^PD-1^+^ ratio in patients with high (n=5) or low (n=12) endogenous GC levels. **(C, D)** Kaplan-Meier survival curves for progression-free and overall survival according to the baseline endogenous GC levels and the proportion of cell subgroups.

We next focused on the impact of endogenous GC levels on the proportion of peripheral blood immune cells. As shown in **Figure 6A**, the proportion of CD8^+^ T cells and CD8^+^PD-1^+^ T cells was similar in two groups, but a low proportion of CD45^+^ cells (p=0.005), CD4^+^ T cells (p=0.048), and NK cells (p=0.031) more commonly occurred in patients with high GC levels, and these patients also had a high proportion of PD-1^+^ NK cells (p=0.012). Furthermore, GC levels were negatively correlated with the ratio of CD8^+^PD-1^+^ to CD4^+^PD-1^+^ ratio (p=0.031) (**Figure 6B**). We also combined two variables including GC levels and specific cell subgroups to determine whether they could better predict clinical outcome for ICI-treated patients. Patients with both high GC levels and high proportion of CD4^+^PD-1^+^ T cells or PD-1^+^ NK cells had a particularly worse prognosis in OS and PFS than other groups of patients (**Figures 6C, D**).

### The endogenous GC and tumor infiltrating lymphocytes

**Figure 7:**
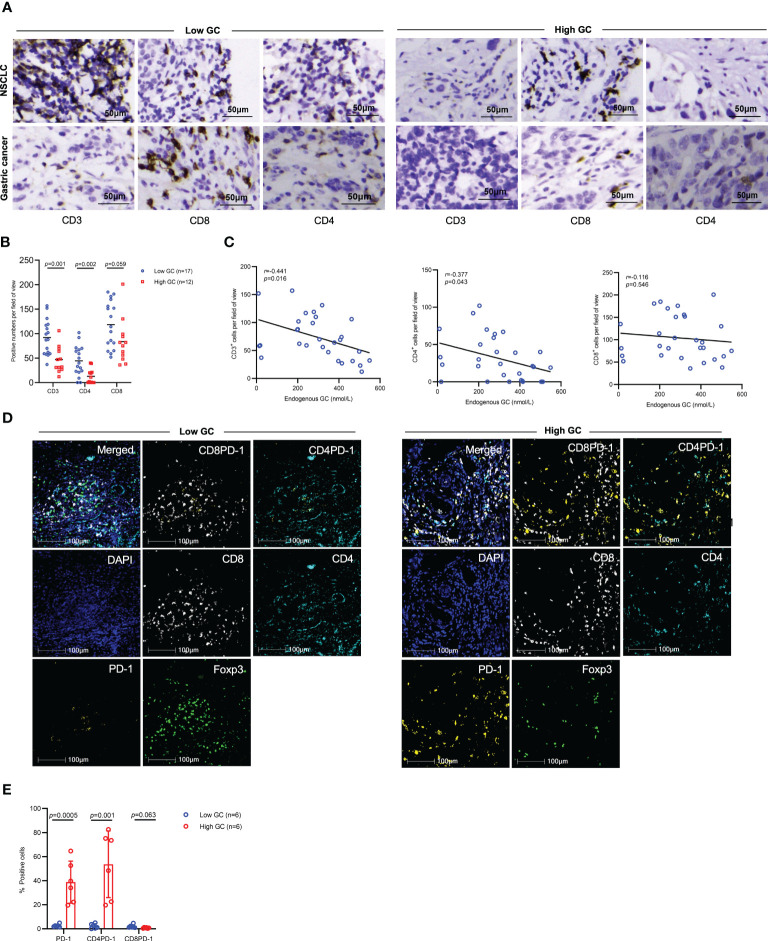
The effect of endogenous GC on immune cell infiltration. **(A)** Representative IHC images for tumor infiltrating CD3^+^, CD8^+^, and CD4^+^ cells in advanced gastric cancer or NSCLC patients with high or low baseline endogenous GC levels. **(B, C)** Comparing the numbers of tumor infiltrating CD3^+^, CD8^+^, and CD4^+^ cells per field of view in advanced cancer patients with high (n=12) or low (n=17) endogenous GC levels. **(D, E)** The mIF analysis of immune cell infiltration in NSCLC tumors with high (n=6) or low (n=6) baseline endogenous GC levels.

We next determined whether endogenous GC levels influenced tumor-infiltrating lymphocytes (TILs) in the tumor microenvironment. In pretreated tumor samples, IHC results showed that patients with high levels of GC had lower numbers of tumor infiltrating CD3^+^ (p=0.001), CD8^+^ (p=0.059), and CD4^+^ (p=0.002) cells compared to those with low levels of GC (**Figures 7A, B**). The levels of GC were inversely associated with the numbers of infiltrating CD3^+^, CD8^+^, and CD4^+^ cells (**Figure 7C**). Furthermore, mIF results showed that patients with high levels of GC had high numbers of PD-1^+^ and CD4^+^PD-1^+^ cells in the tumor microenvironment, but the numbers of CD8^+^PD-1^+^ cells were comparable in two groups (**Figure 7D, E**).

## Discussion

In this study, the endogenous GC levels were found to be higher in advanced cancer patients than that in early-stage cancer patients and healthy individuals. Baseline endogenous GC had a comprehensive negative effect on T cell-mediated immunity and response to immune checkpoint blockade accompanied with cancer progression, which can be developed as a useful biomarker to predict the efficacy and prognosis for advanced cancer patients with immunotherapy. Baseline evaluation of GC should be considered to select potential beneficial cancer populations from ICI therapy.

The anti-inflammatory, anti-shock, and immunosuppressive prospective of exogenous GC are clearly determined (10). GC is routinely utilized to relieve cancer-related dyspnea, fatigue, lack of appetite, and edema. It is also used as first-line agent to combat irAEs associated with immune checkpoint blockade (16, 17). Previous studies show that baseline use of GC (≥10 mg of prednisone equivalent daily) or early use of GC (<2 months after starting immunotherapy) are associated with decreased ORR, PFS, and OS in NSCLC and other solid cancers administrated with PD-1/PD-L1 blockers (18–21, 33). A large systematic review and meta-analysis confirmed the association of steroids use with decreased OS and PFS in patients that received steroids for supportive care or brain metastases (34). In contrast to exogenous GC, the abnormally high levels of plasma endogenous GC (hypercortisolemia) and/or an altered secretion rhythm usually occurs in cancer patients and is associated with advanced stage and poor outcome (25–29). Colorectal cancer or NSCLC patients exhibits an increase in circulating cortisol levels, compared to age- and sex-matched healthy volunteers (24). Plasma levels of cortisol also are significantly elevated in patients with pancreatic ductal adenocarcinoma compared to healthy volunteers (35). Here we comprehensively compared the endogenous GC levels in healthy individuals versus pan-cancer patients with different disease stages. Results showed that not only the abnormality of endogenous GC levels were more likely to occur in advanced cancer patients, but also the endogenous GC levels in advanced cancer patients were higher than that in early-stage cancer patients and healthy populations. The majority of patients with advanced cancer also had lymphopenia and increased NLR, suggesting that the abnormally high levels of peripheral blood endogenous GC were associated with immunosuppression. Thus, the individual’ psychological condition can influence the cells of the immune system, especially if the diagnosis of cancer has already been made. The anxiety and depression will worse clinical outcome for advanced patients due to subsequent continuous therapeutic treatments or rapid progression of the disease itself, accompanied with the increase of systematic GC (35).

Why advanced cancer patients have an abnormally high levels of endogenous GC? Data show that increased endogenous GC secretion is a response to physical and psychological stress-induced hyperactivity of HPA axis. Cancer-related stress induces GC excess, subverts anticancer therapy-induced immunosurveillance, and abolishes therapeutic control of tumors by Tsc22d3 upregulation and significant impairment of the antigen presentation pathway (24). In chronic unpredictable mild stress (CUMS) induced depressed mice models, the progression of liver cancer was significantly accelerated in the depressed mice, and high levels of GC were observed in both depressed mice and depressed HCC patients (15). In the present study, we did not present the stress status of advanced patients, especially those with high levels of endogenous GC. However, the levels of GC were significantly associated with the levels of ACTH, and patients with baseline high levels of endogenous GC remained the high levels of endogenous GC until the first efficacy evaluation regardless of the response to immunotherapy, suggesting that abnormally high levels of endogenous GC are durable and could be controlled by hyperactivity of HPA axis. In addition, endogenous GC can be produced by activated tumor-associated monocyte-macrophage in the tumor microenvironment, and metastatic adrenal gland lesions (36–40).

The mechanism underlying the effect of endogenous GC on the efficacy of immune-dependent cancer therapies remained unclear. Studies indicate that the excessive release of GC promotes PD-1/PD-L1 mediated exhaustion of infiltrated NK cells in the tumor microenvironment accompanied by cancer progression (15). Stress hormone GC is the natural agonist of GR in humans, and its increase during breast cancer progression leads to the activation of the receptor and increased expression of kinase ROR1 at distant metastatic sites, increases colonization, and reduces survival in mice models (41). Endogenous GC surge can subvert anticancer therapy-induced immunity and abolish therapeutic control of tumors in animal models through blocking type I IFN responses in dendritic cell and IFN-γ+ T cell activation, accompanied with cancer progression (24, 42). Even GC presenting in the tumor microenvironment together with IL-12, IL-15, and IL-18 induces the *de novo* expression of PD-1 on NK cells, which are associated with a strong immunosuppressive phenotype (43). The endogenous GC produced by tumor-associated monocyte-macrophage lineage cells regulate effector differentiation and development of dysfunction in CD8^+^ TILs *via* active GR signaling, and results into the failure to respond to checkpoint blockade. The negative impact of local endogenous GCs on tumor microenvironment and immunity can partially explain why adrenal glands acts as immune-resistant sanctuary sites of metastases in renal cancer, melanoma, uterine carcinosarcoma, and even MSI-high metastatic colorectal cancer with immunotherapy (37–40). The expression loss of the antigen presenting genes might be related to the presence of GCs in metastatic adrenal tumors (39). In particular, in adrenocortical carcinoma, GC excess is significantly associated with CD3^+^CD4^+^ T cell depletion, and patients with GC excess and low TILs had a particularly poor overall survival compared to those with normal GC levels and high TILs (27 *vs* 121 months) (44).

Exogenous GC has been showed to suppresses the function of activated T lymphocytes by enhancing expression of PD-1 and other immune checkpoint molecules, and modestly impact the efficacy of checkpoint blockade combination treatment in cellular and animal experiments (45, 46). Fucà and colleagues demonstrated that early use of steroids was associated with worse clinical outcomes and remarkable modulation of peripheral blood immune cells and increased NLR, which could contribute to restraining the activation of antitumor immunity (47). In contrast to exogenous GC use, stress-induced durable augmentation in the endogenous GC tonus inhibits IFN-γ expression in tumor-infiltrating T cells and decreases plasma concentration of cytokines and chemokines, such as IFN-β, IL-15, IL-23A, CXCL10, CXCL1 and CXCL9, and these changes were also long-lasting. Thus, endogenous GC can lead to intra-tumoral and systemic durable immunosuppression (24). In agreement with these clinical findings, we showed that a low proportion of peripheral blood CD45^+^, CD4^+^, NK, CD4^+^PD-1^+^ T, PD-1^+^ NK cells more commonly occurred in patients with high GC levels than those with low GC levels. The ratio of CD8^+^PD-1^+^ to CD4^+^PD-1^+^ was negatively with the circulating levels of GC. Patients with both high GC levels and high proportion of immunosuppressive CD4^+^PD-1^+^ T cells or PD-1^+^ NK cells had a particularly worse prognosis than other groups of patients. These results were also in line with previous reports that circulating naïve PD-1^+^ PBMC, CD4^+^PD-1^+^ T cells, and the balance between circulating CD8^+^PD-1^+^ and CD4^+^PD-1^+^ were negatively correlated with survival or benefit from immune checkpoint blockers in advanced NSCLC (48–50). We also found that tumors with high levels of GC had lower number of TILs compared to those with low levels of GC. Less number of PD-1^+^ and CD4^+^PD-1^+^ cells in the tumor microenvironment were observed in tumor with high levels of GC. Aston et al. found that dexamethasone treatment caused substantial lymphodepletion in peripheral blood but not tumor in patients and mice (46), even low doses of external dexamethasone at the initial stage of treatment or other anti-inflammatory pretreatment is beneficial to improve the efficacy of anti-cancer treatment by counteracting tumor-immunostimulation, suppressing or limiting tumor evasion (11, 12, 51, 52). However, this was not completely consistent with our findings showing that the intermediate levels of endogenous GC are not more beneficial than both higher and lower levels of GC. One explanation may be that exogenous GC treatment has more impact on naïve T cell proliferation and differentiation that are a key source of secondary anti-tumor immunity mediated by antigen spread in response to ICI (13), but influence of endogenous GC on T cell proliferation, differentiation, and function in both peripheral blood and tumor is long-lasting, accompanied with cancer progression and GC secretion in cancer patients.

Interestingly, we also found that circulating IL-6 levels were associated with the levels of GC. Previous investigations indicate that baseline serum IL-6 levels or its changes have been found to be associated with the efficacy of immune checkpoint blockade therapy in NSCLC by altering peripheral T cell population and function (53–55). Targeting IL-6 by genetic ablation or pharmacological inhibition in combination with CD40 stimulation or PD-1/PD-L1 signaling blockade improves T-cell infiltration into tumor and enhances mouse survival (56, 57). Even IL-6 blockade in cancer patients treated with immune checkpoint blockade is viewed as a win-win strategy because combined IL-6 blockade and immune checkpoint blockade abrogates immunotherapy toxicity and promotes tumor immunity (58). Thus, durable high levels of GC during cancer progression could abolish the efficacy of immune-dependent cancer therapies through changing the cancer cell and immune cell-containing microenvironment, the proportion of circulating immune cells, circulating IL-6 levels, and the balance between circulating CD8^+^PD-1^+^ and CD4^+^PD-1^+^ T cells.

Although such real-world data on the effect of baseline GC is possible, our study still has several limitations. First, this study was retrospective with limited overall patient size. Objective response was only determined by direct review of scans by local radiologists and evaluated by RECIST instead of other immune-related response valuation criteria. We only used subgroup analyses in a single cohort, and thus, further external cohort validation for specific cancer type is needed. Second, plasma cortisol concentrations were only analyzed in samples collected only at one time of day (8:00 AM). In fact, the identification of fluctuations of cortisol, namely circadian rhythms, requires a more careful evaluation of the HPA axis levels over a daily period. Third, data on traditional predictive biomarkers, such as PD-L1 expression or TMB, were not available in the majority of patients in this analysis. Fourth, available blood PBMC and tissue samples with flow cytometry or IHC analyses were limited, which may have increased selection bias. Some patient parameters such as pain, sleep dysregulation, and psychological stress or depress levels, were not incorporated to the analysis, which may have influenced our results.

## Conclusions

Our study reports a comprehensive picture of the negative systemic immunomodulatory effects of endogenous GC in cancer patients. We also present a compelling case for caution in considering the initiation of ICI therapy if a patient has high baseline levels of GC. Further prospective trials with larger patient cohorts sampled is needed, and further experimental investigations should be implemented to validate the mechanisms associated with endogenous GC’s effects on function and signaling of circulating immune cell subpopulations, cancer cell itself, and tumor-infiltrating immune cells. More importantly, studies focusing on reversing the adverse influence of endogenous GC on immunity and immunotherapy, such as controlling cancer-related depress and chronic stress with agents, combined IL-6 blockade, and interrupting the GR signaling with targeted molecules, are urgently expected.

## Data availability statement

The original contributions presented in the study are included in the article/**Supplementary Materials**, further inquiries can be directed to the corresponding author/s.

## Ethics statement

The studies involving human participants were reviewed and approved by The First Affiliated Hospital of Shandong First Medical University (NO: YXLL-KY-2020–007). The patients/participants provided their written informed consent to participate in this study.

## Author contributions

All authors contributed in their order in writing the manuscript. YC, XH, HTL, and BW wrote this manuscript and prepared the figures. QX, YG, JC, BY, JX, DF, XW, JiL, JC, XiaL, XinL, WN, LM. HRL, JuL, and YL collected clinical data. HL performed the pathological analysis. JW conceived, wrote this manuscript and is the corresponding author. All authors read and approved the final manuscript.
