# Supplementary material for: Impact of endogenous glucocorticoid on response to immune checkpoint blockade in patients with advanced cancer
Source: Front Immunol. 2023 Apr 11;14:1081790. doi: 10.3389/fimmu.2023.1081790 (PMC10126286; doi:10.3389/fimmu.2023.1081790)
Supplement: Supplementary file 1 [file DataSheet_1.docx]

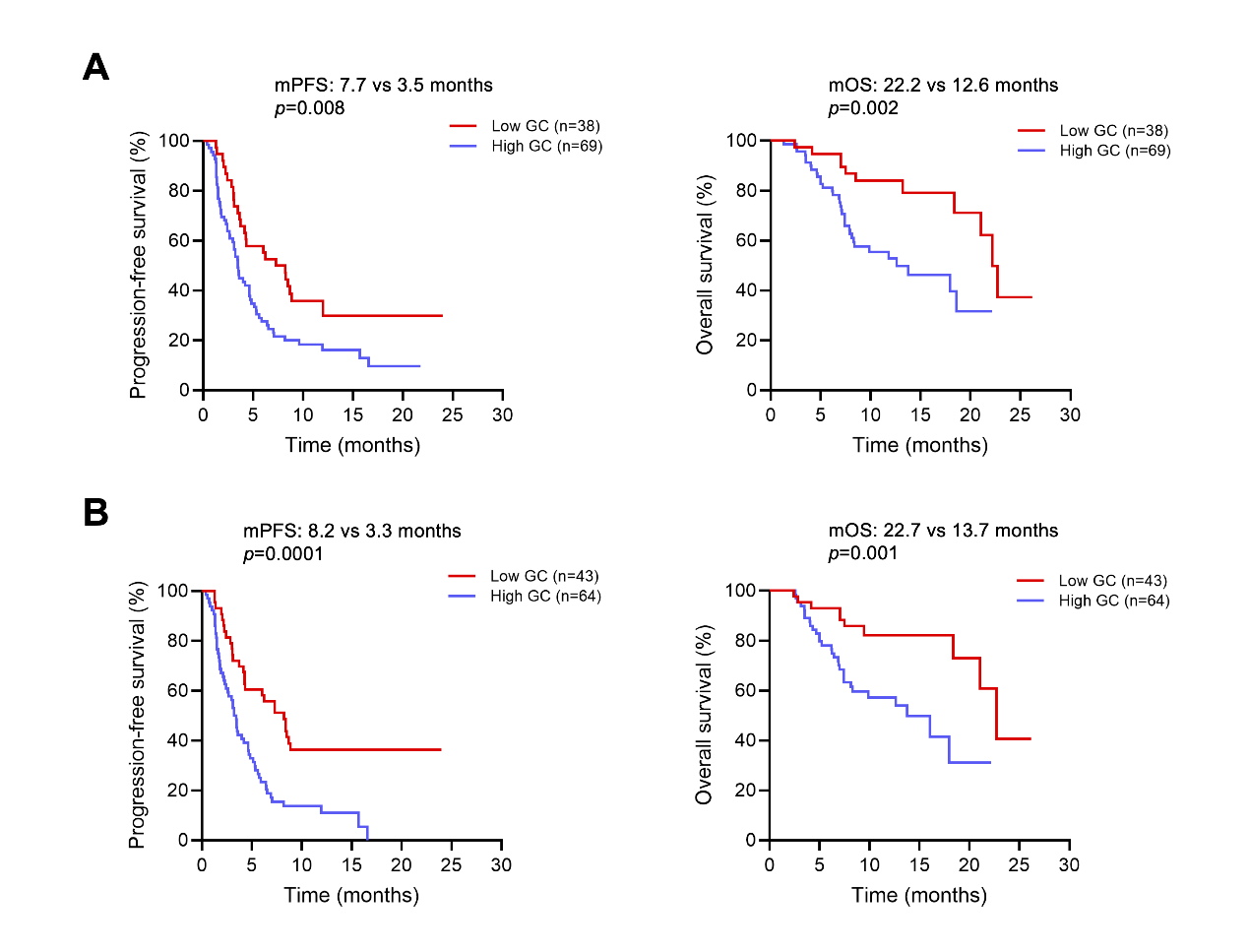


**Supplementary Figure 1** Kaplan-Meier survival curves for progression-free and overall survival according to the baseline endogenous GC levels in patients with anti-PD-1 antibody treatment (**A**, n=107) and combination therapy (**B**, n=107). PSM: propensity score matching; GC: glucocorticoid; NSCLC: non-small cell lung cancer; mOS: median overall survival time; mPFS: median progression-free survival.
