# Supplementary material for: Impact of endogenous glucocorticoid on response to immune checkpoint blockade in patients with advanced cancer
Source: Front Immunol. 2023 Apr 11;14:1081790. doi: 10.3389/fimmu.2023.1081790 (PMC10126286; doi:10.3389/fimmu.2023.1081790)
Supplement: Supplementary file 2 [file DataSheet_2.docx]

**
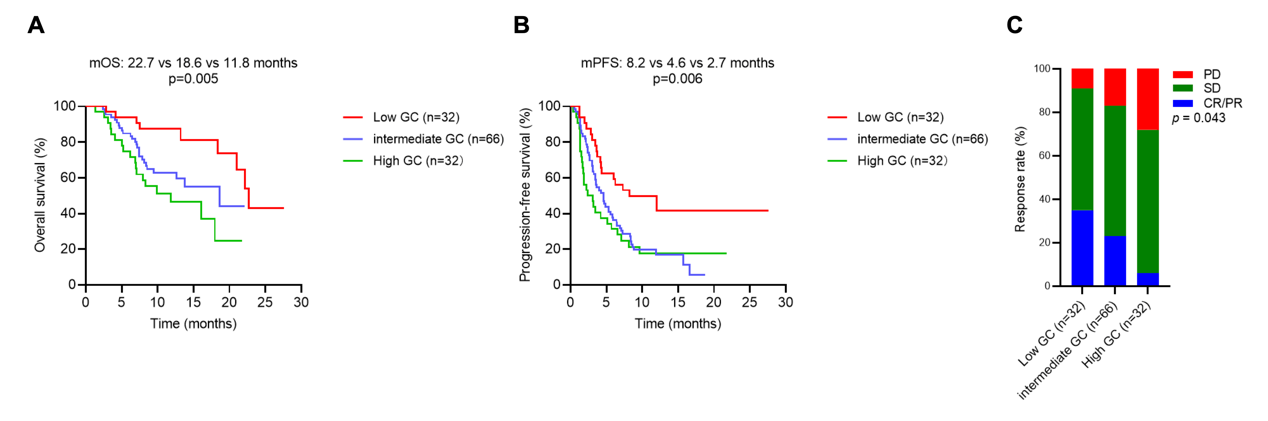
**

**Supplementary Figure 2** Survival and efficacy analysis according to the baseline endogenous GC levels in three groups (low, intermediated, and high levels of GC). Kaplan-Meier survival curves for overall survival (**A**) and progression-free survival (**B**) according to the baseline endogenous GC levels. ORR for advanced cancer patients with high baseline endogenous GC levels versus intermediate and low endogenous GC levels (**C**). GC: glucocorticoid; mOS: median overall survival time; mPFS: median progression-free survival; CR: complete response; PR: partial response; ORR: objective response rate; SD: stable disease; PD: progressive disease.
